# PROTOCOL: Performance pay and employee health: A systematic review

**DOI:** 10.1002/cl2.1272

**Published:** 2022-08-09

**Authors:** Karsten Albæk, Tine Jeppesen, Bjørn Christian Arleth Viinholt

**Affiliations:** ^1^ VIVE—The Danish Center for Social Science Research Copenhagen Denmark

## Abstract

This is the protocol for a Campbell systematic review. The main objective of the review is to answer the following research question: What is the effect of performance pay on employee health?

## 1 BACKGROUND

### 1.1 Description of the condition

Workers can be remunerated based on the amount of time they work (e.g., by a fixed hourly wage) or by the value they produce. The latter is termed performance pay, as it directly links performance to pay. Performance pay can take several forms including individual and group piece rates, where pay is directly linked to the amount of output produced, as well as various kinds of bonuses and profit sharing. Common to the different types of performance pay is that they provide an economic incentive for workers to increase efforts and thus aligns the interests of workers and firms. For this reason, performance pay is often associated with higher productivity and earnings (see e.g., Lazear, 2018).

However, this type of compensation scheme has also been associated with a number of disadvantages, including potential adverse health consequences. These disadvantages were recognised many years ago. In 1776, Adam Smith wrote, ‘Workmen … when they are liberally paid by the piece, are very apt to overwork themselves, and to ruin their health and constitution in a few years’ (Smith, 1776, p. 83).

Work related health problems, whether physical or mental, could have large human and financial costs. First and foremost for the individuals concerned and their families, but also for employers and society at large in terms of productivity and tax losses as well as increased health care costs.

In the UK alone, HSE statistics show that over a million workers are injured or made ill by their work each year (Health and Safety Executive, 2019). It is further estimated that the total cost of (new cases) of work‐related ill health and injuries ran to a total sum of £15 B in 2017/2018. The majority of this was borne by the individuals affected (£8.6 B), while the remaining costs were more or less equally borne by employers (£3.0 B) and government (£3.4 B) (Health and Safety Executive, 2019). For the US, Leigh (2011, p. 729), assesses that the costs of occupational injury and illness in the US was approximately $250 bill. in 2007, which was ‘… at least as large as the cost of cancer’.

### 1.2 Description of the intervention

Performance pay has a long history. Bryson et al. (2012, p. 1), state that ‘Remunerating workers “by the piece” was said by Adam Smith (1976) to be the rule in industry in the 18th century’. The authors also state that ‘Various types of performance bonus schemes, and plans in which firms shared profit with employees …. existed at least since the 1840s in France, the UK and the US …’.

The use of traditional piece rates in industry in advanced economies appears to be limited according to the evidence in Hart (2016). However, other forms of performance pay is widely used.

On the basis of household surveys Bryson et al. (2012) investigate the prevalence of performance pay in the US (the General Household Survey for 2002 and 2006) and various European countries (the European Working Conditions Survey for 2000 and 2005). These surveys provide detailed information on individual bonuses and piece rates, profit/gain sharing and share ownership schemes. The authors restrict attention to employees with a permanent contract, employed in private sector, in profit‐oriented firms only and do not consider managers and CEOs. Bryson et al. (2012) find substantial cross‐country differences in the share of permanent employees in the private sector receiving some form of performance‐related pay: From between 10% and 15% in Portugal, Greece, Belgium, Germany, UK and Ireland to over 40% in Sweden, Finland and the US. During the period there was an increase in both individual performance pay, profit gainsharing and share ownership in both the US and in the European countries (Bryson et al., 2012, p. 4).

In both Europe and the US, the prevalence of profit/gain sharing schemes and share ownership is greater in high‐skilled occupations than in low‐skilled occupations (e.g., one‐digit group two, professionals, in the International Standard Classification of Occupation, in in contrast to one‐digit group 9, elementary occupations). In Europe, individual‐level incentives, including piece rates, are more concentrated among those in low‐skilled occupations, whereas in the US those in high‐skilled jobs are more likely to have such schemes.

The interventions considered in this review are performance pay, where workers are paid by the amount they produce, in contrast to pay per hour, where workers are paid according to their time input. Performance payment can take several forms including piece rates, commissions, various kinds of bonuses and profit sharing. Furthermore, performance pay can be tied to performance at the individual level and performance at the group level.

In our review, we will include all types of performance pay and will note the type being analysed in each study. This will, however, not always be possible as the literature often relies on surveys to uncover the use or receipt of performance pay by individual firms (Dahl & Pierce, 2020), or employees (Artz & Heywood, 2015; Bender & Theodossiou, 2014), and do not always distinguish between the different types of performance pay. We will apply a separate coding for those studies, where it is not possible to identify the precise type of performance pay for all the workers that are included in the study.

#### 1.2.1 The rationale for the intervention

The standard rationale for a firm adopting performance pay as a remuneration scheme is to increase the productivity and profit of the firm. Potential adverse health effects could be considered as side effects, whose magnitude is not expected to be so large that the firm abstain from adopting performance pay. While the positive impact of performance pay on profit accrues to the firm, the major share of the potential cost of adverse health effects is expected to fall upon workers and society at large (e.g., in the form of increased health care costs).

#### 1.2.2 Usage of performance pay

The literature on performance pay include theoretical models that apply economic theory to investigate the consequences of the choice for firms and workers (Barth et al., 2006; Lazear, 1995; Prendergast, 1999). The literature investigates the topic under various assumptions. A typical set up is a firm that can choose to remunerate workers either by a fixed salary or by performance pay. With a fixed salary, a worker is payed for the amount of input (time) and the remuneration does not depend on the output produced by the worker. Performance pay is modelled such that the remuneration of a worker consists of a fixed component and a share of the output that the worker produces. The ability and the effort of the worker is assumed to be unobservable or not directly observable. The output of a worker is assumed to be observable when costly monitoring is used or the output is assumed to be indirectly observable via a signal of the output. The workers’ contribution to revenue depends on the skills of the worker, the effort of the worker and the outcome of a random event.

Typical predictions from these type of model are that workers are more likely to be employed in a firm with performance payment than in a firm with fixed salary payment when:

- The cost for workers of supplying more effort is low (this could be expected for high ability workers or high skilled workers (Barth et al., 2006; Lazear, 2000)
- The workers' risk aversion is low (Barth et al., 2006; Lazear, 1995; Prendergast, 1999)

Firms are more likely to adopt performance pay when:

- The marginal monitoring cost of worker is low (Lazear, 1995)
- The noise in the output signal is low (Barth et al., 2006; Prendergast, 1999)

Compared to workers in fixed salary firms workers in firms with performance pay earn:

- Higher wages on average (Lazear, 2000; Prendergast, 1999)
- More dispersed wages (Lazear, 2000)

Predictions from stylised economic models can in turn be applied to establish predictions about health outcomes for workers with performance payment compared to workers with fixed salary. These effects can—in theory—be both positive and negative.

### 1.3 How the intervention might work

Health effects from performance pay can occur through a number of channels including:

- *Increased workload/work pace*:
  o Under performance pay wages depend directly on the performance of either the individual employee or a group of employees. This provides an incentive for individual employees to increase efforts including their workload and work pace. This can directly cause both physical and mental health problems such as stress, exhaustion, musculoskeletal disorders, pains, accidents and injuries (Bender & Theodossiou, 2014; Johansson et al., 2010). An increased workload can also indirectly cause a number of adverse health conditions, if it results in longer working hours and thereby leaves less time to spend on healthy activities (Bender & Theodossiou, 2014).
- *Increased risk taking*
  o In some jobs, performance pay can also provide an incentive for employees to take greater physical risks, which in turn can lead to an increased rate of accidents (Johansson et al., 2010). In addition, physical health problems arising as a result of performance pay, whether due to increased risk taking or an increased workload, can in turn generate or exacerbate mental health problems (Dahl & Pierce, 2020).^1^
- *Change in income level*
  o The adoption of performance pay is expected to increase the average income level of workers. Increased income can lead to better health through more purchase of goods and services that are expected to further health, for example healthy food, fitness activities and services from doctors and hospitals. Positive health effects of performance pay can thus arise through the income channel. However, while adoption of performance pay is expected to increase the average income among workers, lower performing employees might experience lower income, with possibly adverse effects on health (Sweet et al., 2013). Performance pay might thus have a heterogeneous impact on employee health across the distribution of worker productivity.
- *Increased uncertainty and variance of income*
  o Performance pay can affect health through income in several ways. First, there is an increased uncertainty associated with the level of income under performance pay, which can lead to stress (Bender & Theodossiou, 2014). Second, performance pay gives rise to a greater variance in pay across workers with similar jobs. This can in turn induce anxiety and depression among all but the top‐performing employees (Dahl & Pierce, 2020).
- *Uncooperative work environment*
  o Performance pay can also impact negatively on the mental health of employees by intensifying competition between employees (Dahl & Pierce, 2020). Chan et al. (2013) provide evidence indicating that this type of behaviour is especially promoted by performance pay that rewards individual performance.

Both negative and positive health consequences arising from performance pay can in turn impact other outcomes including the degree of absence from work, the use of medicine and health care resources (e.g., doctor visits, emergency department use) and may also influence the degree to which employees are likely to take up early retirement (Szubert & Sobala, 2005).

For workers who experience adverse health consequences from performance payment, the impact on absence from work is ambiguous. On the one hand, absence may increase due to illness (Devaro & Heywood, 2017). On the other hand, performance pay increases the value of being present despite being ill or injured, and absence may thus be reduced (Dale‐Olsen, 2012).

Some of the potential negative health outcomes of performance pay might be counteracted by institutions or traits of society. For example, the income effect on health may be smaller in countries with universal health care systems compared to countries where some workers are not covered by health insurance. In the same vein, health consequence of uncertainty of income may be smaller in countries with more generous support schemes for workers who experience reductions in income due to unemployment or illness. Furthermore, stricter and more strictly enforced safety regulations, which are likely to vary across countries, might in some cases reduce the potential negative consequences of performance pay. Therefore the country in focus in each of the studies will be included in the data for the review. If the number of studies in different countries are sufficiently large, we will attempt to make a moderator analysis aiming at investigating whether variables for universal health care systems, generosity of income support and strictness of safety regulations have consequences for the magnitude of adverse impacts of performance pay on health.

**Figure 1:**
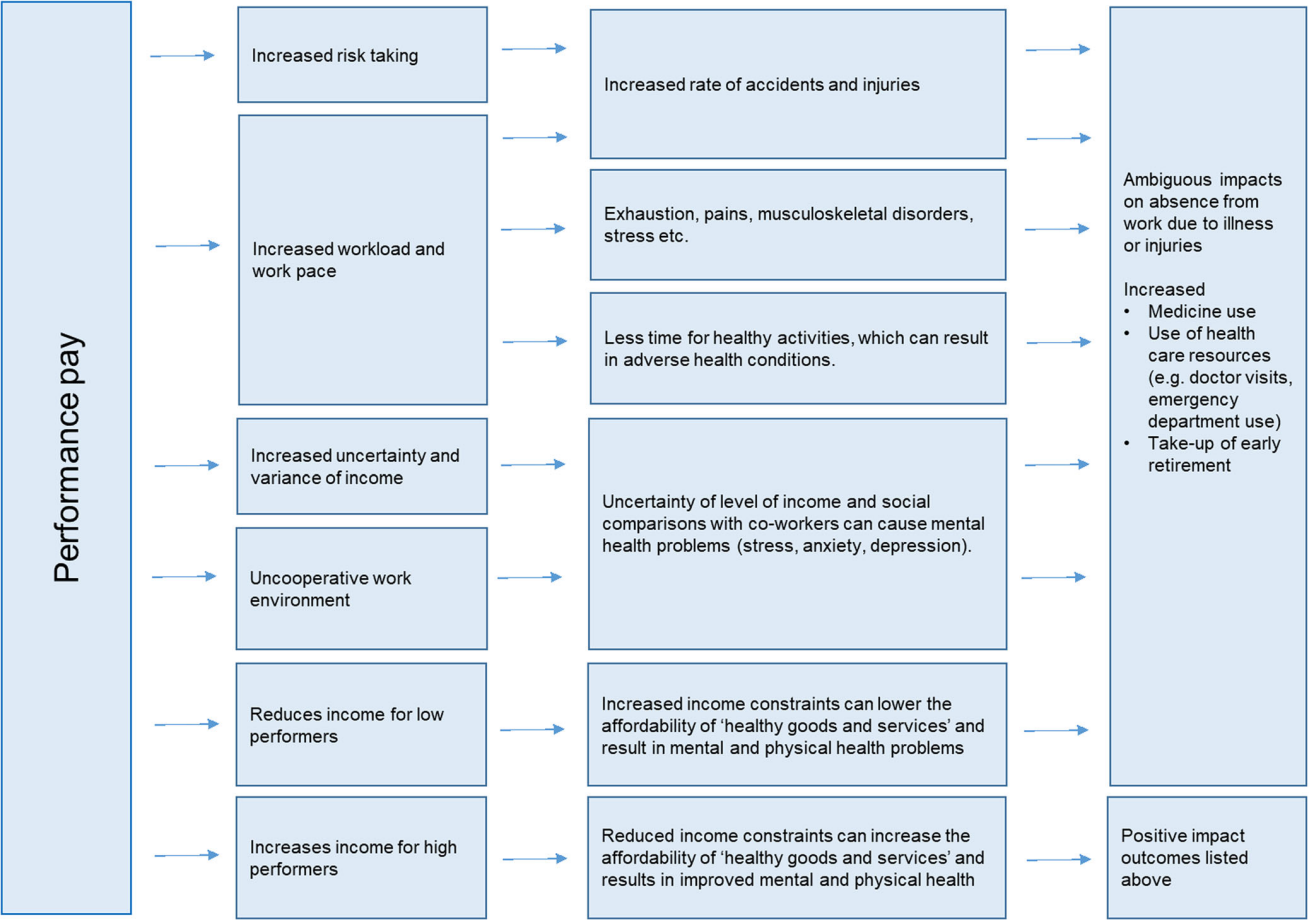
Channels through which performance pay can affect health

Figure 1 provides an overview of the different channels discussed above.

### 1.4 Why it is important to do this review

Performance pay plays an important role in remuneration of workers in many countries. There is evidence that the prevalence of performance pay is rising, especially for higher skilled occupations. A substantial literature exists, which shows that performance pay can be beneficial for firms in the form of higher productivity and for workers in the form of higher wages (e.g., Lazear, 2000).

However, if performance pay has unintended negative consequences for the health of workers, and the costs of deteriorated health are only partly covered by firms and workers, a substantial share will be incurred by the society at large. This is one of the rationales for interventions in the form of legislation with respect to working environment, which in Europe take place both at the national and at the European Union level. Occupational health and safety regulations have the potential to decrease detrimental consequences of performance pay on health. The review findings will thus be of interest from a policy point of view, for workers, employers and the society at large.

#### 1.4.1 Prior reviews

Remuneration systems (including performance pay) are one out of many ways by which firms can affect employee well‐being and performance of firms. Van De Voorde et al. (2012) reviews the literature of the impact of Human Resource Management (HRM), defined as ‘all those activities associated with the management of people in firms’, which besides performance pay includes recruitment and selection, training and development, performance management, teamwork, employment security, participation and communication. The authors trace the impact of HRM on three measures of employee well‐being including health (the other two are ‘happiness’ and ‘relationships’) and two measures of organisational outcomes (‘operational outcomes’ and ‘financial outcomes’).^2^

The review by Van De Voorde et al. (2012) includes seven studies that analyse the relationship between HRM and health‐related well‐being and find that the majority of the data points show a negative relationship. This is in contrast to the finding of mostly positive relationships between HRM and the other two measures of employee well‐being, ‘happiness’ and ‘relationships’ (the findings of the studies included in the review are summarised by ‘vote‐counting’).

To the best of our knowledge, the valuable review Johansson et al. (2010) is the only existing review whose primary focus is on the relation between performance pay and health. The authors review articles published up to 2008. The number of articles included in their review is 31 and, of these, 27 find that piece rate pay has negative health consequences.

The authors state that the criteria for including studies in the review were relevance and quality. More specifically (Johansson et al., 2010, p. 608) write: ‘Articles and reports that dealt with health and safety aspects in a superficial or marginal way were excluded from further examination.’ Furthermore, the authors state that ‘An important quality criteria was the type of publication the research was published in…’, and ‘Quality was also judged by looking at the described and used research methodology and the general impression of the performed and reported research.’

The authors do not seem to discuss the role of control groups in assessing whether a study should be included in the review. The review includes a study of accidents in the construction industry that does not contain a control group (Gravseth et al., 2006). The review furthermore include a study (Brisson et al., 1992) that compares health outcomes for textile workers, among which half were on piece rate, with outcomes for administrative workers.

For all studies assessed in the review, a qualitative statement of the finding is included. While odds‐ratios are reported for some studies, the authors do not undertake a summary calculation of the overall effect found in the studies. Furthermore, the studies included in the review are concentrated in specific industries, especially transportation, textile, and foresting. Finally, the review does not seem to include articles published in economics journals. Thus, while the findings of the review support the hypothesis of a negative relation between piece rates and health, there appears to be scope for a re‐evaluation of the relationship between performance pay and health using more stringent inclusion criteria and including newer studies.

Our review will differ from the previous review (Johansson et al., 2010) in a number of ways. First, the previous review focuses only on piece rate pay, whereas we will focus more broadly on performance pay. Second, since the above review was undertaken a number of new studies have appeared that seems to come closer to establishing a causal link from performance pay to health (some of these have been published in economics journals). Third, we will apply the more stringent criteria of the Campbell Collaboration for inclusion of studies in the review, especially with respect to the presence of a control group in the studies. Fourth, we intend to present overall estimates of the impact of performance pay on health by doing meta‐analysis on the effects reported in the studies included in the review. Fifth, we expect that our review will have a broader industry scope as some recent studies have utilised data spanning across industries.

#### 1.4.2 Description of methods used in primary research

The studies included in the review will have variables that measure the health of employees as the outcome variable. The outcome variables are measured for a group of workers, who are remunerated by performance pay (the intervention group), and a group of workers, who are not on performance pay but are remunerated according to time input to the production process of firms (the control group). A standard procedure is variants of regression models where the left hand side is a health variable and the right hand side consists of an indicator for performance payment and various control variables.

The data are typically collected by surveys where either workers or management of firms answer questions on the type of employee payment. The analysis include an assignment of workers in the surveys to either an intervention or a control group. The surveys are typically not collected from randomised experiments (or trials), and the studies are thus ‘observational studies’. Randomised experiments try to ensure that the intervention and the control group are similar with respect to observed and unobserved characteristics. Such an equivalence is, however, less likely to be the case in observational studies, which may impact on the measured relationship between performance payment and health.

For example, if workers on performance pay are more likely to take risks than workers paid by time input, then a part of excess health problems among the intervention group might be attributed to high risk taking behaviour among this group (i.e., present irrespective of the type of remuneration for this group). The difference in health problems between the intervention and the control group thus consists both of the effect of performance pay on health and an effect that is a consequence of the difference in characteristics between the intervention and the control group. Even if there are no discernible differences between observed characteristics (such as age and gender) between the intervention and the control group, there might be differences between the (unobserved) propensity to take risk. Note that a prediction of theoretical models on performance pay is that individuals with low propensity to take risks will select into jobs without performance payment.

According to the theory, workers with high ability (or skills) and low risk aversion are more likely to be employed in firms with performance pay. To take this into account, some studies include proxy variables for these characteristics (e.g., Artz & Heywood, 2015) who applies an aptitude test as a proxy for ability and a variable for smoking as an indication of risk aversion). Some studies account for differences in risk aversion by analysing subgroups that potentially differ in the degree of risk aversion, especially by analysing males and females separately. The general perception is that females are more risk averse than men, where the evidence stems mostly from experimental economics, although Filippin (2016) claims that gender difference might not be so large and robust as commonly assumed).

A remedy for the problem of unobserved differences between the intervention and the control group is present in studies where the same group of workers is followed before and after changes in the remuneration system. In this case, the effect of performance pay on health is obtained as the change in health outcome before and after the change in remuneration system for the same group of workers. If workers' propensity to take risks does not change over time, then using the same group of workers as both intervention and control group will result in estimates of the impact of performance payment on health that are not confounded from differences in unobserved differences in the propensity to take risks. However, health for a group of workers can change over time for other reasons than a change in the remuneration system. In this case, the change in health before and after a change in remuneration system will thus be a biased estimate of the impact of performance payment on health to the extent that health changes over time for other reasons than the change in remuneration system.

The problem that other factors than the remuneration system can have an impact on changing health outcomes over time is addressed in studies using data with both an intervention group (whose remuneration system changes over time) and a control group (whose remuneration system does not change over time). In these studies, the effect of performance pay is obtained as the difference in the change over time in health outcomes between the intervention group and the control group. In this case, the impact of other factors will only confound the estimate of the impact of performance pay on health to the extent that these factors have a different impact on the intervention and the control group over time.

#### 1.4.3 Description of representative studies illustrating methods

Artz and Heywood (2015) use survey data from six waves of the US National Longitudinal Study of Youth from 1988 to 2000 to estimate the impact of performance pay on the risk of illness or workplace injury. The sample size is 36,900 worker‐year observations. Five types of performance pay are included in the survey: Piece rates, bonuses, commissions, stock options and tips. The critical performance pay indicator equals 1 if a worker receives either piece rates, performance bonuses or both, and 0 if the worker receives neither. The authors attempts to account for the effect of self‐selection by applying an aptitude test as a proxy for ability and a variable for smoking as an indication of risk aversion. They furthermore control for unobserved time invariant employee characteristics via fixed effects estimation on the panel of workers. Based on the information in the article it is possible to convert the following estimates to effect sizes (coefficients divided by standard deviation): the risk of injury or illness for the whole sample of workers and for the subsamples of blue‐collar workers and white‐collar workers. To convert other results (e.g., for the subsamples of male and female workers) to effects sizes, we plan to contact the authors.

Frick et al. (2013) analyse consequences of a transition in a large German steel company from hourly pay to performance pay and teamwork from 1994 to 2000. Workers became organised in ‘production units’ (consisting of 80–300 members) of which some received performance pay in the form of a joint bonus, some became organised in teams and others had both teamwork and performance pay. The authors perform panel data analysis on monthly observations for production units and contrast outcomes with the outcomes for hourly pay. The number of monthly observations varies according to the analysed outcome measure: From 5088 for accidents, 3180 for absence rates and 2200 for productivity. The information in the article makes it possible to calculate effect sizes for absence rates and productivity (output and adjusted output, which is output adjusted for material waste that workers do not take into account when performance pay is based on output). With respect to accidents, the authors do not present the results in a way that makes it possible to calculate effect sizes and we will therefore contact the authors to obtain the necessary information.

## 2 OBJECTIVES

The main objective of the review is to answer the following research question: What is the effect of performance pay on employee health?

Further, the review will attempt to answer the questions:

- Do the effects differ among different groups of employees such as different age classes and gender?
- Do the consequences of performance pay differ between the different types of health measures, such as muscle/skeleton problems, psychic problems, absenteeism?
- Do the effects on various health measures differ between the length of exposure to performance pay and do effects differ with respect to the time it takes to develop health problems?
- Do the effects differ with respect to the type of performance pay, such as piece rates, individual performance pay, or group performance pay?
- Do the effects differ between industries and occupational groups?
- Do the effects vary across countries or country groups with different institutional characteristics that might have an impact on the health effects of performance pay?

Finally, the review will try to assess the degree of gains from performance pay (as secondary outcomes for studies that investigate health consequences of performance pay) in the form of:

- Wage increases for workers
- Improved firm performance (e.g., productivity and financial outcomes)

## 3 METHODS

### 3.1 Criteria for considering studies for this review

#### 3.1.1 Types of studies (research methods/designs)

To summarise what is known about the possible causal effects of performance pay, we will include all study designs that use a control group of workers not paid by performance (paid by the hour or salaried).

The study designs we will include in the review are:

1. Randomised and quasi‐randomised controlled trials (allocated at either the individual level or cluster level, for example, groups of workers within firms).
2. Non‐randomised studies (performance pay has occurred in the course of usual decisions, the allocation to performance pay and no performance pay is not controlled by the researcher, and there is a comparison of two or more groups of participants, that is, at least a treated group and a control group).

A requirement for all types of studies (randomised as well as non‐randomised) is that they are able to identify an intervention effect. Cross‐sectional studies where, for example, the treatment is given to workers in one firm only and the comparison group is workers in another firm (or more firms for that matter) cannot separate the treatment effect from the firm effect.

One debatable issue is if studies that investigate the consequences of introduction (or abandonment) of performance pay in one firm should be included in the review. There are prominent examples of one‐firm studies in the economic literature on the effects of performance pay on wages and productivity, for example, Freeman and Kleiner (2005) analyse the consequences of abandoning performance pay in a shoe manufacturing firm and Lazear (2000) trace the effects of introducing performance pay in a firm in the automobile repair business. If all workers in the firm are lumped together, the control group is the workers in the firm before the change in remuneration system. These types of ‘single group pre‐post comparisons’ are, with due reason, excluded in reviews in many areas. However, in some cases some workers in the firm experience changes in the remuneration system while others do not. In these cases intervention and control groups can be constructed at the same point of time and valid analysis of the effect of the intervention can be conducted under the assumption that changes in the environment affects both the intervention and the control group in a similar way (the aforementioned study Frick et al. (2013) is an example of this type of analysis). In our review we will exclude results for single firms that are based on single group pre‐post comparisons but include results from studies of single firms if they compare the outcome for workers in intervention and control groups at the same point of time.

#### 3.1.2 Types of participants

The ‘intervention population’ is adult employees in all industries and occupational groups. This implies that we will exclude self‐employed persons as well as children (most countries have an age limit of 18 before a worker is considered adult). Finally, we will also exclude studies that focus on CEOs (we will exclude studies, where inclusion of CEOs might have any noticeable impact on the results).

#### 3.1.3 Types of interventions

The interventions considered in this review are all types of performance pay. This includes all pay schemes that compensate employees based on their individual or collective performance. The contrast to this is payment schemes that compensate employees exclusively based on their time input (e.g., workers paid per hour or salaried workers paid per week or month). The intervention is typically decided by employers but may be decided after consultation with the employees of the firm.

In the review, we will include studies where performance pay makes up all or part of the compensation. We will include information on the share of the pay that is made up by performance pay if this information is contained in the study. The comparison group is workers paid per hour or salaried workers (in some studies the comparison group might comprise workers on different payment system and the inclusion criteria for the studies is that there is a substantial difference between the share of workers on performance pay in the intervention group and the control group). If the information in the studies makes it possible, we intend to construct an ordinal scale indicating the extent of performance pay in the studies in the review [e.g., from Partial (under 50%), Substantial (over 50%–99%) to Fully (100%)].

#### 3.1.4 Other criteria

We will include interventions in all types of firms across all industries in both the private and public sector in all countries.

#### 3.1.5 Types of outcome measures

##### Primary outcomes

The primary outcomes in the study are measures related to the health of workers. These outcomes include:

- Work related accidents and injuries
- Physical and mental health outcomes (e.g., musculoskeletal problems, premature ageing, stress, anxiety, depression, etc.)
- Medicine use
- Use of health care resources (e.g., doctors' visits, emergency department use)
- Sickness absence (or absenteeism) from the work‐place
- Early retirement from the labour market (i.e., retirement before the typical retirement age)
- Physiological effects (e.g., levels of measured adrenaline)

##### Secondary outcomes

Secondary outcomes are

- Workers'
  o Job satisfaction
  o Earnings
  o Productivity
- Firm (or plant)
  o Productivity
  o Profit
  o Financial status

###### Duration of follow‐up

Performance pay can impact health via a number of channels, some of which may be independent of the duration of time (e.g., the risk of accidents and injuries) and some of which are increasing in the length of time paid using performance pay (e.g., stress, physical ailments due to increased workload/work pace). It is therefore relevant to include studies that measure both the short and long‐term impacts of performance pay on health.

The review will include analyses of workers where the intervention still take place (work under performance pay) and workers where the intervention is terminated (work under performance pay has stopped). We will make no restrictions based on either the duration of exposure or the duration of follow‐up, but will note both of these in the data extraction table, to the extent possible. To the extent that the information is available, we plan to use both time of exposure and time of follow up as moderator variables (portioned in suitable time periods).

### 3.2 Search methods for identification of studies

Relevant studies will be identified through searches in electronic databases, governmental and grey literature repositories, hand search in specific targeted journals, citation tracking, contact to international experts and Internet search engines.

#### 3.2.1 Electronic searches

##### Electronic databases

The following electronic databases will be searched:

- Academic Search (EBSCO)
- CINAHL (EBSCO)
- EconLit (EBSCO)
- International Bibliography of the Social Sciences (ProQuest)
- PsycINFO (EBSCO)
- Sociological Abstracts (ProQuest)
- Science Citation Index Expanded (Web Of Science)
- Social Sciences Citation Index (Web Of Science)

##### Description of search‐string

The search string is based on the PICO(S)‐model. We identified four aspects of the topic, and developed a search facet for each. All of the four facets are searched in title, abstract and subject terms (according to the options of each database). An example of the search string as it will be implemented on the database Academic Search is seen below:

**Table:** 

Search	Terms	Results
S17	S4 AND S8 AND S12 AND S16	(1228)
S16	S13 OR S14 OR S15	(10,296,451)
S15	DE ‘RANDOMIZED controlled trials’ OR DE ‘CONTROL groups’ OR DE ‘EXPERIMENTAL design’ OR DE ‘CASE‐controlmethod’	(155,484)
S14	AB (random* OR control* OR group* OR trial* OR effect* OR experiment* OR pretest* OR posttest* OR ‘case stud*’ OR ‘panel data’ OR ‘repeated measure*’ OR observational*)	(9,798,064)
S13	TI (random* OR control* OR group* OR trial* OR effect* OR experiment* OR pretest* OR posttest* OR ‘case stud*’ OR ‘panel data’ OR ‘repeated measure*’ OR observational*)	(2,283,815)
S12	S9 OR S10 OR S11	(6,516,385)
S11	DE ‘HEALTH’ OR DE ‘MENTAL health’ OR DE ‘HEALTH behavior’ OR DE ‘MUSCULOSKELETAL system injuries’ OR ‘ACCIDENTS’ OR DE ‘WOUNDS & injuries’ OR SU Musculoskeletal*	(410,089)
S10	TI (health* OR disease* OR safe* OR accident* OR injur* OR ill* OR musculoskeletal* OR ‘medic* use*’ OR stress* OR sick* OR absen* OR workload* OR risk* OR ‘early retire*’ OR ‘premature aging’)	(1,674,573)
S9	AB (health* OR disease* OR safe* OR accident* OR injur* OR ill* OR musculoskeletal* OR ‘medic* use*’ OR stress* OR sick* OR absen* OR workload* OR risk* OR ‘early retire*’ OR ‘premature aging’)	(6,085,159)
S8	S5 OR S6 OR S7	(798,661)
S7	AB (employe* OR worker* OR laborer* OR labourer*)	(748,664)
S6	TI (employe* OR worker* OR laborer* OR labourer*)	(76,283)
S5	DE ‘EMPLOYEES’	(51,129)
S4	S1 OR S2 OR S3	(62,931)
S3	AB (‘Performance pay’ OR ‘piece rate*’ OR ‘piece work’ OR ‘piece wage*’ OR incentive*)	(54,790)
S2	TI (‘Performance pay’ OR ‘piece rate*’ OR ‘piece work’ OR ‘piece wage*’ OR incentive*)	(8701)
S1	DE ‘PAY for performance’ OR DE ‘PERFORMANCE awards’ OR DE ‘INCENTIVE awards’ OR DE ‘PIECEWORK’ OR DE ‘EMPLOYEE bonuses’ OR DE ‘WAGE payment systems’ OR DE ‘PRODUCTIVITY incentives’ OR DE ‘LABOR incentives’ OR DE ‘MONETARY incentives’	(11,646)

- Search 1–3 in the search string covers the intervention
- Search 5–7 covers the population
- Search 9–11 covers the outcome
- Search 13–15 covers the study types/methodology

These four facets are combined in the final search on each database (S17 in the example). The search fields covering the subject terms (S1, S5, S11 and S15) will be modified accordingly to the controlled vocabulary on each database.

Limitations of the search‐string

We do not intend to apply a time or language limitation on the database searches.

#### 3.2.2 Searching other resources

##### Hand‐Search

We will conduct a hand search of the following journals to make sure that relevant articles are found. The hand search will focus on editions published between 2016 and 2020 to secure recently unpublished articles, which have not yet been indexed in the bibliographic databases.

- ILR Review
- Economica
- Oxford Economic Papers
- Labour Economics

##### Searches for unpublished literature in general

For the sake of transparency, we have split the search strategies for each type of unpublished literature. In general, most of the resources searched for this purpose includes multiple types of literature. As an example, the resources listed to identify reports from national bibliographical resources also include working papers and dissertations, as well as peer‐reviewed references. Google searches will be utilised for different purposes, but for the sake of simplicity, it is only listed once as a resource.

##### Search for dissertations

We will search the following resources for dissertations:

- ProQuest Dissertations & Theses Global (ProQuest)
- EBSCO Open Dissertations (EBSCO‐host)

Further resources for identifying dissertations might be added during the search process. A final list of resources will be included in the appendix of the review.

##### Search for working papers/conference proceedings

We will search the following resources for working papers/conference proceedings:

- NBER Working Papers—http://www.nber.org/papers.html
- IZA ‐ Institute of the Study of Labor—www.iza.org
- EconPapers (RePEc)—https://econpapers.repec.org/scripts/search.pf
- Open Grey—http://www.opengrey.eu/
- Google Scholar—https://scholar.google.com/
- Google searches—https://www.google.com/
- Social Science Research Network—https://www.ssrn.com/index.cfm/en/

Further resources for identifying working papers and conference proceedings might be added during the search process. A final list of resources will be included in the appendix of the review. Google scholar and general Google searches will be limited to the top 100 hits, sorted by relevance.

##### Search for reports and non‐US literature

We will search the following resources for reports and *non‐US literature*:

- Danish National Research Database—http://www.forskningsdatabasen.dk/en
- SwePub—Academic publications at Swedish universities—http://swepub.kb.se/
- NORA—Norwegian Open Research Archives—http://nora.openaccess.no/
- CORE—research outputs from international repositories—https://core.ac.uk/

Further resources for identifying reports might be added during the search process. A final list of resources will be included in the appendix of the review.

##### Search for systematic reviews

We developed a specific search string to identify other systematic reviews in the databases listed above. This was done simultaneously with the development of the search‐string described above, and the identified relevant reviews are considered in the content of this protocol.

We will also search for systematic reviews on the following resources:

- Campbell Journal of Systematic Reviews—https://campbellcollaboration.org/
- Cochrane Library—https://www.cochranelibrary.com/
- Centre for Reviews and Dissemination Databases—https://www.crd.york.ac.uk/CRDWeb/

##### Citation‐tracking

We will use citation‐tracking methods to identify more literature that is relevant. We will use both citation‐track forwards (by using Google Scholar and Web of Science) and backwards (by screening citations in the most relevant literature).

##### Contact to experts

We will contact international experts to identify unpublished or ongoing studies.

### 3.3 Data collection and analysis

#### 3.3.1 Selection of studies

Under the supervision of the review authors, two review team assistants will first independently screen titles and abstracts to exclude studies that are clearly irrelevant. Studies considered eligible by at least one assistant, or studies where there is insufficient information in the title and abstract to judge eligibility, will be retrieved in full text. The full texts will then be screened independently by two review team assistants under the supervision of the review authors. Any disagreement of eligibility will be resolved by the review authors. Exclusion reasons for studies that otherwise might be expected to be eligible will be documented and presented in an appendix.

The study inclusion criteria will be piloted by the review authors (see Supporting Information: Appendix 1). The overall search and screening process will be illustrated in a flow diagram. None of the review authors will be blind to the authors, institutions, or the journals responsible for the publication of the articles.

#### 3.3.2 Data extraction and management

Two review authors will independently code and extract data from included studies. A coding sheet will be piloted on several studies and revised as necessary (see Supporting Information: Appendix 2). Disagreements will be resolved by consulting a third review author with extensive content and methods expertise. Disagreements resolved by a third reviewer will be reported. Data and information will be extracted on available characteristics of participants, intervention characteristics and control conditions, research design, sample size, risk of bias and potential confounding factors, outcomes, and results. Extracted data will be stored electronically. Analysis will be conducted using Stata software.

#### 3.3.3 Assessment of risk of bias in included studies

We do not expect to find any studies using a randomised controlled trial to examine the impact of performance pay on health. However, in the event that we do find such a study, we will assess the risk of bias using the latest version of the Cochrane risk of bias tool (ROB 2) (Sterne et al., 2019).

To assess the risk of bias in non‐randomised studies we will use the Risk Of Bias in Non‐randomised studies of Interventions (ROBINS‐I) tool, developed by members of the Cochrane Bias Methods Group and the Cochrane Non‐Randomised Studies Methods Group (Sterne et al., 2016). We will use the latest template for completion (currently it is the version of 19 September 2016).

The ROBINS‐I tool covers seven domains through which bias might be introduced into non‐randomised studies:

1. Bias due to confounding
2. Bias in selection of participants
3. Bias in classification of interventions
4. Bias due to deviations from intended interventions
5. Bias due to missing data
6. Bias in measurement of the outcome
7. Bias in selection of the reported result

The tool contains a set of signalling questions to facilitate the judgement about the risk of bias in each domain, which in turn is mapped into an overall judgement of the risk of bias of the study as a whole. Given the intervention and controls meet the criteria for selection for the review we intend to include the study and indicate differences in effect size as a function of degree of bias.

At least two review authors will independently assess the risk of bias for each included study. Any disagreements will be resolved by a third reviewer with content and statistical expertise and will be reported. We will report the risk of bias tables for each included study in the completed review

##### Confounding

We are mainly interested in the effect of starting and adhering to the intended intervention, that is, the treatment on the treated effect and confounding is a potentially threat for identifying this effect. An important part of the risk of bias assessment of non‐randomised studies is consideration of how the studies deal with confounding factors, that is, factors that predict both outcome and treatment (Sterne et al., 2016). This issue is also highly relevant in non‐randomised studies focusing on performance pay, as workers self‐select into these types of jobs. Systematic baseline differences between individuals compensated by performance pay and individuals compensated by an hourly rate may thus compromise comparability between the two groups.

Based on enterprise and household data respectively, Barth et al. (2006); and Bryson et al. (2012) both examine the prevalence of performance pay, and find gender and education to be among the determining factors. Barth et al. (2006) find that enterprises that use performance pay schemes tend to be characterised by a higher share of college‐educated workers and a lower share of female workers, while Bryson et al. (2012) find that female employees are less likely to be compensated by performance pay, while the opposite is true for employees in high‐skilled occupations.

A number of studies have furthermore shown a positive relation between education and health—a relationship known as the ‘education gradient’—which is partly explained by differences in health behaviour, such as smoking, obesity and heavy drinking (Cutler & Lleras‐Muney, 2010). Similarly, gender is an important determinant of mental health, with women being twice as common as men for being depressed (WHO, 2000; p. 31).

Risk preferences also matter, as less risk averse employees may be more attracted to performance paid jobs, as shown by a number of economic models (e.g., Barth et al., 2006), while also being less likely to engage in activities to mitigate the risk of, for example, accidents or injuries (Artz & Heywood, 2015).

Finally, failure to control for industry differences may also result in biased estimates, as both the use of performance pay and work‐related health risks vary across industries. One reason that performance pay is not randomly distributed across industries is that most types of performance pay require easy monitoring of each employee's performance. If this is easier to do in jobs that are inherently dangerous (e.g., due to the operation of heavy equipment), this is a crucial aspect to consider to avoid biased estimates of performance pay on health outcomes (Artz & Heywood, 2015).

Based on our pre‐existing knowledge of the subject matter, we have identified the following confounding factors to be most relevant: Education, gender, industrial affiliation, occupation (e.g., white‐ and blue‐collar workers), and risk preferences. In each study, we will assess whether these factors have been considered either by design of the study or by inclusion of explicit controls. In addition, we will assess other potential factors likely to be a source of confounding within the individual included studies.

#### 3.3.4 Measures of treatment effect

We will report an index of the direction and magnitude of performance pay on each health outcome of interest reported in the study. For ease of comparison of magnitudes across outcomes, we plan to report the same index for all outcomes. Furthermore, reporting the same index for all studies makes it possible to calculate overall (average) measures of the impact of the intervention for the different outcomes for all studies that enter the review.

The common index will either be the standardised mean difference in the form of Cohen's *d* or the correlation coefficient. Cohen's *d* is probably the most common effect size measure in social science. However, some reviews of the impact of Human Resource Management on firm and worker outcomes apply the correlation coefficient as effect size measure (e.g., Crawford et al., 2010; Judge & Piccolo, 2004; Nielsen et al., 2017). A comparison of the results of this review with the results of these types of reviews might thus be easier if effect sizes are reported as correlation coefficients. In the following, we describe how effects sizes in the studies are obtained and presented in the Cohen's *d* metric. The potential conversion to the correlation coefficient metric will take place using the standard formula for this conversion.^3^

We expect that most studies included in the review report results in the form of coefficients obtained by applying variants of regression analysis. The effect size that enters the review will typically be calculated from a regression, where the estimate of the effect of performance pay on health is adjusted by inclusion of various covariates or confounders as explanatory variables.

In the standard case of a continuous outcome variable (e.g., average number of days absent among a group of workers), the effect size becomes the coefficient on the treatment variable divided by the standard deviation of the outcome variable. The standard deviation of the outcome variable is either obtained from the descriptive statistics included in the studies or from the standard deviation of the residuals of a regression, where only the treatment variable enter as an explanatory variable. In at least one study, which is expected to be included in the review, the outcome variable is normalised by dividing by the standard deviation. In this case, the coefficient on the treatment variable is an estimate of the effect size.

In cases where studies apply a dichotomous outcome variable (e.g., an individual having experienced a work‐related accident or illness), it is likely that the results from several procedures are going to enter our review. In a linear probability model, the coefficient on the treatment variable is analogous to the coefficient when the outcome variable is continuous and enter as the numerator of the effect size measure. If the standard deviation of the outcome variable is not included in the descriptive statistics, we will calculate it from the mean of outcome variable as the variance of a dichotomous outcome variable can be calculated from the mean.

Alternative procedures for estimating a regression model with a dichotomous outcome variable include the probit and the logit model. In some cases, the results of applying these models are reported as ‘marginal effects’. These effects correspond to the coefficients obtained using a linear regression model is employed. The effect size will thus be calculated as the marginal effect of the treatment variable divided by the standard deviation of the outcome variable. Results obtained using the logit model are often reported in the form of odds ratios. In these cases we will transform odds ratios to Cohen's d using the methods suggested by Sánchez‐Meca et al. (2003).

Some studies might not apply regression analysis but report results directly from tabulations of outcomes for treatment and control groups. For continuous outcomes, we will calculate effects sizes from means and standard deviations, when these are available. If means and standard deviations are not available, we will calculate standardised mean differences from *F*‐ratios, *t*‐values, *χ* ^2^ values and correlation coefficients, where available, using the methods suggested by Lipsey and Wilson (2001). For dichotomous outcomes, we will transform odds ratios or risk ratios to Cohen's d using the methods suggested by Borenstein et al. (2009).

For each reported effect size in our report, we will present an estimate of the standard error of the effect size. We will do this by applying the standard formula for the variance of the standardised effect size obtained in Hedges (1981) and replicated in, for example, Hedges and Olkin (1985, p. 81, eq. 10). The entities entering the formula is Cohen's *d* and the size of the treatment and the control group. In some regression analyses, more than one treatment group enters (e.g., performance pay only and teamwork only). In these cases, we will apply the number of units in the reference group of the regression as the control group size.

Regression models in different studies often apply various various covariates: the case of no covariates result in ‘bivariate effect sizes’ while the inclusion of covariates results in ‘partial effect sizes’. If both type of effect sizes appear in our survey, we will follow the advice in Aloe et al. (2016) and make separate meta‐analyses for each type of effect size. According to Aloe and Thompson (2013); each partial effect size in a meta‐analysis may be estimating a different population parameter, suggesting that the random‐effects model is more appropriate than a fixed effect model. We thus intend to use the random‐effects model when synthesising the partial effect sizes in the studies included in our survey.

#### 3.3.5 Unit of analysis issues

Errors in statistical analysis can occur when the unit of allocation differs from the unit of analysis. In cluster randomised trials, participants are randomised to treatment and control groups in clusters, either when data from multiple participants in a setting are included (e.g., creating a cluster within the firm), or when participants are randomised by for example treatment locality. Non‐randomised studies may also include clustered assignment of treatment. Effect sizes and standard errors from such studies may be biased if the unit‐of‐analysis is the individual and an appropriate cluster adjustment is not used (Higgins & Green, 2011).

If possible, we will adjust effect sizes individually using the methods suggested by Hedges (2007) and information about the intra‐cluster correlation coefficient (ICC), realised cluster sizes, and/or estimates of the within and between variances of clusters. If it is not possible to obtain this information, we will adjust effect sizes using estimates from the literature of the ICC (e.g., Hedges, 2007), and assume equal cluster sizes. To calculate an average cluster size, we will divide the total sample size in a study by the number of clusters.

#### 3.3.6 Dealing with missing data

We expect that many studies do not report standard effect size measures that can enter the data synthesis of the intervention. However, a fair share is expected to contain the sufficient information to make the calculation of standard effect size measures possible. If a study does not contain sufficient data to allow calculation of effect size estimates authors will be contacted to obtain necessary summary data, such as means and standard deviations or standard errors. In cases where the information cannot be obtained from the authors, we will assess to what extent it appears credible to apply estimates of the missing pieces of information from other studies included in the review.

#### 3.3.7 Assessment of heterogeneity

As the interventions of interest deal with diverse populations of participants (from different countries, in different occupations and industries, etc.), we expect heterogeneity among primary study outcomes. For each outcome category, we will provide a graphical display (forest plot) of effect sizes and the associated confidence intervals.

The forest plot will display the effect sizes in an order such that the oldest studies (oldest with respect to the year the performance pay took place) will be placed in the top of the plot and the youngest in bottom. This ordering will make it possible to get a graphical overview of the extent to which the effect of performance pay on health has changed over time (some health effects could have diminished over time, for example, because of the use of safer machinery, while others may have increased over time).

Heterogeneity among primary outcome studies will be assessed with *χ* ^2^ (*Q*) test, and the *I* ^2^, and *τ* ^2^ statistics (Higgins et al., 2003; or Deeks et al., 2008). The *χ* ^2^ test has low power when studies have small sample sizes, but we expect this problem to be of minor magnitude in the present study, as sample sizes are likely to be larger than in many other reviews. We plan to use the software Revman 5 for the analyses (but might shift to Stata or R if appropriate).

#### 3.3.8 Assessment of reporting biases

We will use funnel plots for information about possible reporting bias if we find a sufficient number of studies. One source of reporting bias (or non‐reporting bias) is publication bias that can be present if studies that attain significant findings are more likely to be published than studies that do not obtain significant results. However, asymmetry in funnel plots can be caused by other reasons than reporting bias, such as differences in methodological quality and heterogeneity among studies (Page et al., 2020).

#### 3.3.9 Data synthesis

The analysis of the effect sizes will take place in a couple of steps. First, we will group the measures of the effect sizes according to the outcome category (absence from work place, work place accidents, etc.). For each outcome category, we will present the effect sizes and the associated 95% confidence intervals from the different studies in displays. Given the displays indicate heterogeneity among the included studies, In the assessment of the overall effect for the different outcome categories we will thus apply inverse variance weighting using random effects statistical models that incorporate both the sampling variance and between study variance components.

Furthermore, we will attempt to give an overall assessment of the effect of performance pay on the health of workers across different health measures. That is, we analyse effects sizes combined or pooled across different types of outcome. The a priori hypothesis is that performance pay potentially has an adverse impact on workers' health and we will only combine outcomes for which this hypothesis is relevant or credible. The following outcome measures will thus not be included in the combination of outcomes:

- Secondary outcomes (worker pay and productivity)
- Absence rates (absence is not a health outcome but absences rates are expected to increase if performance pay has a negative impact on health; conversely, the incentive effects of performance pay may decrease absence rates).

In the first meta‐regression of the pooled data set of effect sizes for different outcomes, we will enter categorical variables for outcomes. The result of this joint regression makes it possible assess the extent to which the impact of performance payment on health differ between the various outcome measures and if differences are significantly different.

In the next step, we will enter the moderators and follow the procedure to be described in the next section. The pooled data set with a larger number of effect sizes will make it more likely to discover the potential impact of moderators in the assessment of the impact of performance payment on health.

At the outset, we perform the estimations using standard random effects models, where the difference between the effect sizes is modelled by a variance component, which is assumed to be normally distributed (the default estimation procedure is Restricted Maximum Likelihood).

If effect sizes cannot be pooled, study‐level effects will be reported in as much detail as possible. Software for storing data and statistical analyses will be RevMan 5.0, Excel, R and Stata 10.0.

Our review will probably contain studies that present more than one effect size, that is, the pooled data set consisting of effect sizes from different subgroups will have observations that are not likely to be independent. For example, if a study produces a high effect size for one outcome, effect sizes for other outcomes might also be high. In this case, effect sizes are positively correlated within the studies. Meta‐analyses that do not take this dependence into account, are not likely to yield correct estimates of the variance components and estimates of aggregate measures of effect sizes, as the variance components enter the weights that are applied to calculate average effect sizes.

To address this problem we intend first to estimate models where an additional variance component enter to take within study correlation into account (e.g., Konstantopoulos, 2006 contains an exposition of estimation when effect sizes are correlated). We plan to perform this modelling in R (applying package ‘metafor’).

Second, we intend to to apply robust variance estimation (RVE) approach (Hedges et al., 2010) to address the issue of correlated effect sizes. This method assumes a random‐effects model in which study average effect sizes vary across studies (with variance *τ* ^2^) and the effect sizes within each study are equicorrelated (with correlation coefficient *ρ*), but the method does not assume that the variance components are normally distributed. The method uses approximate inverse‐variance weights, given assumptions about the unknown ρ. We will calculate weights using estimates of *τ* ^2^, setting *ρ* = 0.80 and conduct sensitivity tests using a variety of *ρ* values to asses to which degree the general results and estimates of the heterogeneity is robust to the choice of *ρ*. We will use the small sample adjustment to the residuals used in RVE and the Satterthwaite degrees of freedom (Satterthwaite, 1946) for tests (Tipton, 2015).

The results in (Tipton, 2015) suggests that the degrees of freedom depend on not only the number of studies but also on the type of covariates included in the meta‐regression. The degrees of freedom can be small, even when the number of studies is large if a covariate is highly unbalanced or a covariate with very high leverage is included, The degrees of freedom will vary from coefficient to coefficient. The corrections to the degrees of freedom enable us to assess when the RVE method performs well. As suggested by Tanner‐Smith and Tipton (2014); and Tipton (2015) if the degrees of freedom are smaller than four, the RVE results should not be trusted.^4^

#### 3.3.10 Subgroup analysis and investigation of heterogeneity

We will attempt to apply meta‐regression to the effect sizes presented in our study. The moderators are explanatory variables that potentially have an impact on the direction and magnitude of the effect sizes. A priori, there are reasons to expect that several moderators potentially have health consequences for workers on performance pay. We intend to investigate the role of the following pre‐specified moderators (given sufficient information in the primary studies about these variables):

- Gender
- Age (most likely in age categories)
- Occupation (most likely in a binary variable for blue‐ and white‐collar workers)
- Industry (dummy variables for different industries)
- Length of exposure of performance pay
- Time of measurement after exposure
- Type of performance pay (piece rate, bonuses, etc.)
- Unit of performance pay (payments to individuals or to groups)
- Research design (RCTs vs. non‐RCTs)
- Countries and regions (an assessment of the potential impact of welfare regimes of different countries is likely beyond the scope of the study).

It is almost completely certain that it will be impossible to say something about the joint effect of these 10 moderators. If they are all applied to an outcome, for which 10 effect sizes are reported, the level of the effect sizes will be ‘explained’ exactly and statistical assessment of the validity of the regression model is not possible.

We will thus proceed entering the moderators stepwise. Initially we will enter each of the moderators alone in meta‐regressions and report the results. Then we will report results when more than one moderator enter the regressions. The decision of which moderators to enter jointly will depend on the significance of the coefficients in the first step and the correlation between the different moderators.

#### 3.3.11 Sensitivity analysis

Sensitivity analysis will be carried out by restricting the meta‐analysis to a subset of all studies included in the original meta‐analysis and will be used to evaluate whether the pooled effect sizes are robust across components of risk of bias. We will consider sensitivity analysis for each domain of the risk of bias checklists and restrict the analysis to studies with a low risk of bias. If the results vary across different groups, we will evaluate the result when the ROB ratings are used as moderators in meta‐regressions.

##### External validity

Performance payment of various forms takes place in different industries, occupations and countries with varying regulations for the working environment and could potentially have an impact of various dimensions of workers' health. For example, injury rates are typically high in the construction industry and low for white collar workers, and the potential impact of performance pay on injury rates is also likely to exhibit substantial variation. The aim of our review is thus not to come up with an overall assessment of the effect of performance pay on for example injury rates that is valid across different worker groups.

The potential effect of performance pay on the health of different worker groups that are not included in the review will thus have to be evaluated by assessing the similarity to groups of workers that are included in the survey. The aim of the review is to provide relevant effect sizes by aggregating individual effect sizes for different subgroups and, hopefully, to provide a condensed and more precise overview of the effect of performance on workers' health in the form of the results from meta‐regression.

##### Costs

As stated in the introduction to this protocol performance pay is expected to incur costs to workers, to firms and to society. The costs to workers include the monetary costs of the consequences of illness and injuries, such as reduced payments due to absence from work places. The costs to firms include potential increases in absence rates and decreases in the quality of the production (e.g., when increased material waste counteracts increases in output). The costs to society include costs related to worker illness that are not covered by workers and firms (e.g., health care costs, especially in countries with universal health care systems). Our review will provide assessments of the impact of performance pay on workers' health that are relevant for carrying out cost–benefit analyses of performance payment, although we do not plan to includes such analyses in the review

##### Treatment of qualitative research

We do not plan to include qualitative research.

## CONTRIBUTIONS OF AUTHORS

Karsten Albæk, Ph.D. (economics) is an experienced empirical labor economist who has published broadly in international journals, including high‐impact journals, on topics including employment, wage formation, health and working environment. Karsten has conducted a systematic review about the impacts of new technology on the labor market and on the Danish labor market ‘flexicurity’ system. His field of expertise is labor markets, program evaluation, econometrics and statistics (including methodology), and he will contribute to the quantitative data extraction, methodological quality appraisal, and meta‐analysis.

Tine Jeppesen, Ph.D. (economics) is an experienced empirical economist and has worked primarily in the field of international economics. She has contributed to several high‐level policy reports and economic impact assessments of trade agreements and cross border investments, including the impact of foreign direct investments on productivity and employment levels among local firms. Tine's fields of expertise are program evaluation, econometrics, and statistics. She will contribute to the quantitative data extraction, methodological quality appraisal, and meta‐analysis.

Trine Filges, Ph.D. (economics) is an experienced systematic reviewer and methodologist, having completed a number of systematic reviews in social welfare topic areas as well as in the field of education. She has published thirteen Campbell Systematic reviews, is currently the lead reviewer on three Campbell Systematic Reviews, is involved as a reviewer in two additional Campbell Systematic Reviews, and has published systematic and meta‐analytic reviews in high‐impact journals. Trine's fields of expertise are systematic review methods and statistical analysis. She will contribute to the quantitative data extraction, methodological quality appraisal, and meta‐analysis.

Bjørn Christian Arleth Viinholt has 3 years of experience in developing and writing systematic reviews. Bjørn has experience in developing systematic search strategies and processes of reference management. Bjørn will contribute to the development of the systematic search strategy, reference management, and grey literature searches for this review.

- Content: Karsten Albæk and Tine Jeppesen
- Systematic review methods: Karsten Albæk, Tine Jeppesen and Trine Filges
- Statistical analysis: Karsten Albæk and Tine Jeppesen
- Information retrieval: Bjørn Viinholt

## DECLARATIONS OF INTEREST

Karsten Albæk participate in a project aiming at investigating the consequences of performance pay on workers' health in the Danish construction industry. If the project results in a paper before the completion of the review, the other members of the team will assess if results in the paper are to be included in the review.

## PRELIMINARY TIMEFRAME

Approximate date for submission of the systematic review is within 2 years of protocol approval.

## PLANS FOR UPDATING THIS REVIEW

Once completed, we plan to update the review with a frequency of 2 years if funding is available. Karsten Albæk will be responsible.

## SOURCES OF SUPPORT

**Internal sources**

• Campbell group, VIVE—The Danish Center for Social Science Research, Denmark

Financing stems from the Campbell group

• New Source of support, Other

There is no other support

**External sources**

None, Denmark

There is no external support

## Supporting information

Supporting information.

## Footnotes

^1^
Grund (2010) present evidence that German workers with high risk aversion are less likely to be employed in jobs with performance pay. Individual risk attitude is measured on a scale from ‘unwilling to take risk’ to ‘fully prepared to take risk’. This measure has been validated experimentally by showing that it is a good predictor for actual risk taking behaviour in lottery choice experiments. The study is thus evidence for a connection between taking risk in monetary matters (e.g., choosing performance pay), which is the risk concept applied in the theoretical work on performance pay, and risky behaviour at the workplace (e.g., choosing actions that might cause accidents), which is the risk concept that is relevant for some of the health outcomes considered in the empirical studies on the health effects of performance pay.

^2^
The recent contribution Nielsen (2017) reviews workplace resources' impact on employee well‐being (which includes health) and performance, but workplace resources are defined as ‘anything perceived by the individual to help attain his or her goals’ and performance pay is thus not included in this concept.

^3^
The formula is r = *d*/(4 + *d* ^2^)½ where *r* is the correlation coefficient and *d* is Cohen's *d*.

^4^
If we apply robust variance estimation, the analysis will be conducted in STATA or R as robust variance estimation is not implemented in Revman 5.

## References

[cl21272-bib-0001] ADDITIONAL REFERENCES

[cl21272-bib-0002] Aloe, A. M. , & Thompson, C. G. (2013). The synthesis of partial effect sizes. Journal of the Society of Social Work and Research, 4(4), 390–405.

[cl21272-bib-0003] Aloe, A. M. , Tanner‐Smith, E. E. , Becker, B. J. , & Wilson, D. B. (2016). *Synthesizing bivariate and partial effect sizes* (Campbell Methods Series: Policy Note 2).

[cl21272-bib-0004] Artz, B. , & Heywood, J. S. (2015). Performance pay and workplace injury: Panel evidence. Economica, 82, 1241–1260.

[cl21272-bib-0005] Barth, E. , Bratsberg, B. , Hægeland, T. , & Raaum, O. (2006). *Who pays for performance?* (IZA Discussion Paper, No. 2142).

[cl21272-bib-0006] Bender, K. A. , & Theodossiou, I. (2014). The unintended consequences of the rat race: the detrimental effects of performance pay on health. Oxford Economic Papers, 66, 824–847.

[cl21272-bib-0007] Borenstein, M. , Hedges, L. V. , Higgings, J. P. T. , & Rothstein, H. R. (2009). Introduction to meta‐Analysis. John Wiley & Sons.

[cl21272-bib-0008] Brisson, C. , Vezina, M. , & Vinet, A. (1992). Health problems of women employed in jobs involving psychological and ergonomic stressors: The case of garment workers in Quebec. Women & Health, 18(3), 49–65. 1615690 10.1300/J013v18n03_04

[cl21272-bib-0009] Bryson, A. , Freeman, R. , Lucifora, C. , Pellizzari, M. , & Perotin, V. (2012). *Paying for performance: Incentive pay schemes and employees' financial participation* (CEP discussion Paper No. 1112).

[cl21272-bib-0010] Chan, T. Y. , LI, J. , & Pierce, L. (2013). Compensation and peer effects in competing sales teams. Management Science, 60(8), 1965–1984.

[cl21272-bib-0011] Crawford, E. R. , LePine, J. A. , & Rich, B. L. (2010). Linking job demands and resources to employee engagement and burnout: A theoretical extension and meta‐analytic test. Journal of Applied Psychology, 95(5), 834–848. 20836586 10.1037/a0019364

[cl21272-bib-0012] Cutler, D. M. , & Lleras‐Muney, A. (2010). Understanding differences in health behaviors by education. Journal of Health Economics, 29, 1–28. 19963292 10.1016/j.jhealeco.2009.10.003 PMC2824018

[cl21272-bib-0013] Dahl, M. S. , & Pierce, L. (2020). Pay‐for‐performance and employee mental health: large sample evidence using employee prescription drug usage. Academy of Management Discoveries, 6(1), 12–38.

[cl21272-bib-0014] Dale‐Olsen, H. (2012). Sickness absence, performance pay and teams. International Journal of Manpower, 33(3), 284–300.

[cl21272-bib-0015] Deeks, J. J. , Higgins, J. P. T. , & Altman, D. G. (2008). Analysing data and undertaking meta‐analyses. In P. T. Higgins Julian , & Green Sally (Eds.), Cochrane handbook for systematic review of interventions (pp. 243–296). John Wiley & Sons.

[cl21272-bib-0016] Devaro, J. , & Heywood, J. S. (2017). Performance pay and work‐related health problems: A longitudinal study of establishments. Industrial and Labor Relations Review, 70(3), 670–703.

[cl21272-bib-0017] Filippin, A. (2016, May). Gender differences in risk attitudes . IZA World of Labor (Vol. 100, pp. 1–10).

[cl21272-bib-0018] Freeman, R. B. , & Kleiner, M. M. (2005). The last American shoe manufacturers: Decreasing productivity and increasing profits in the shift from piece rates to continuous flow production. Industrial Relations, 44(2), 307–330.

[cl21272-bib-0019] Frick, B. J. , Goetzen, U. , & Simmons, R. (2013). The hidden costs of high‐performance work practices: Evidence from a large German steel company. ILR Review, 66(January), 198–224.

[cl21272-bib-0020] Gravseth, H. M. , Lund, J. , & Wergeland, E. (2006). Risk factors for accidental inuries in the construction industry (Risikofaktorer for ulykkesskader i bygge‐ og anleggsbransjen). Tidsskrift for den Norske Legeforening, 126(4), 453–456. 16477284

[cl21272-bib-0021] Grund, C. , & Sliwka, D. (2010). Evidence on performance pay and risk aversion. Economics Letters, 102, 8–11.

[cl21272-bib-0022] Hart, R. (2016, April). The rise and fall of piecework . IZA World of Labor (Vol. 254, pp. 1–10).

[cl21272-bib-0023] Health and Safety Executive . (2019, March). *Costs to Britain of workplace fatalities, self‐reported injuries and ill health, 2017/18*. Government report. p. 18.

[cl21272-bib-0024] Hedges, L. V. (1981). Distribution theory for Glass's estimator of effect size and related estimators. Journal of Educational Statistics, 6, 107–128.

[cl21272-bib-0025] Hedges, L. V. (2007). Effect sizes in cluster‐randomized designs. Journal of Educational and Behavioral Statistics, 32, 341–370.

[cl21272-bib-0026] Hedges, L. V. , & Hedberg, E. C. (2007). Intraclass correlation in cluster‐randomized designs. Journal of Educational and Behavioral Statistics, 29(1), 60–87.

[cl21272-bib-0027] Hedges, L. V. , & Olkin, I. (1985). Statistical methods for meta‐analysis. Academic Press.

[cl21272-bib-0028] Hedges, L. V. , Tipton, E. , & Johnson, M. C. (2010). Robust variance estimation in meta‐regression with dependent effect size estimates. Research Synthesis Methods, 1(1), 39–65. 26056092 10.1002/jrsm.5

[cl21272-bib-0029] Higgins, J. , & Green, S. (2011). Cochrane handbook for systematic review of interventions. Wiley‐Blackwell.

[cl21272-bib-0030] Higgins, J. , Thomson, S. G. , & Altman, D. G. (2003). Measuring inconsistency in meta‐analyses. Britisk Medical Journal, 327, 557–560. 10.1136/bmj.327.7414.557 PMC192859 12958120

[cl21272-bib-0031] Johansson, B. , Rask, K. , & Stenberg, M. (2010). Piece rates and their effects on health and safety—A literature review. Applied Ergonomics, 41(4), 607–614. 20106469 10.1016/j.apergo.2009.12.020

[cl21272-bib-0032] Judge, T. A. , & Piccolo, R. F. (2004). Transformational and transactional leadership: A meta‐analytic test of their relative validity. Journal of Applied Psychology, 89(5), 755–768. 15506858 10.1037/0021-9010.89.5.755

[cl21272-bib-0033] Konstantopoulos, S. (2006). *Fixed and mixed effects models in meta‐analysis* (IZA Discussion Paper 2198).

[cl21272-bib-0034] Lazear, E. P. (1995). Personal economics. MIT Press.

[cl21272-bib-0035] Lazear, E. P. (2000). Performance pay and productivity. The American Economic Review, 90(5), 1346–1361.

[cl21272-bib-0036] Lazear, E. P. (2018). Compensation and incentives in the workplace. Journal of Economic Perspectives, 32(3), 195–214.

[cl21272-bib-0037] Leigh, P. J. (2011). Economic burden of occupational injury and illness in the United States. The Milbank Quarterly, 89, 728–772. 22188353 10.1111/j.1468-0009.2011.00648.x PMC3250639

[cl21272-bib-0038] Lipsey, M. W. , & Wilson, D. B. (2001). Practical meta‐analysis. SAGE Publications.

[cl21272-bib-0039] Nielsen, K. , Nielsen, M. B. , Ogbonnaya, C. , Känsälä, M. , Saari, E. , & Isaksson, K. (2017). Workplace resources to improve both employee well‐being and performance: A systematic review and meta‐analysis. Work & Stress, 31(2), 101–120.

[cl21272-bib-0040] Page, M. J. , Higgins, J. P. T. , & Sterne, J. A. C. (2020). Chapter 13: Assessing risk of bias due to missing results in a synthesis. In J. P. T. Higgins , J. Thomas , J. Chandler , M. Cumpston , T. Li , M. J. Page , V. A. Welch (Eds.), Cochrane handbook for systematic reviews of interventions . Version 6.1 edition, updated September 2020. Cochrane.

[cl21272-bib-0041] Prendergast, C. (1999). The provision of incentives in firms. Journal of Economic Literature, 37(1), 7–63.

[cl21272-bib-0042] Satterthwaite, F. (1946). An approximate distribution of estimates of variance components. Biometrics, 2, 110–114. 20287815

[cl21272-bib-0043] Smith, A. (1776). An inquiry into the nature and causes of the wealth of nations. PF Collier & Son.

[cl21272-bib-0044] Sterne, J. A. , Hernán, M. A. , Reeves, B. C. , Savović, J. , Berkman, N. D. , Viswanathan, M. , Henry, D. , Altman, D. G. , Ansari, M. T. , Boutron, I. , Carpenter, J. R. , Chan, A. W. , Churchill, R. , Deeks, J. J. , Hróbjartsson, A. , Kirkham, J. , Jüni, P. , Loke, Y. K. , Pigott, T. D. , … Higgins, J. P. (2016). ROBINS‐I: A tool for assessing risk of bias in non‐randomised studies of interventions. BMJ, 355(4919), 1–7. 10.1136/bmj.i4919 PMC5062054 27733354

[cl21272-bib-0045] Sterne, J. A. C. , Savović, J. , Page, M. J. , Elbers, R. G. , Blencowe, N. S. , Boutron, I. , Cates, C. J. , Cheng, H. Y. , Corbett, M. S. , Eldridge, S. M. , Emberson, J. R. , Hernán, M. A. , Hopewell, S. , Hróbjartsson, A. , Junqueira, D. R. , Jüni, P. , Kirkham, J. J. , Lasserson, T. , Li, T. , … Higgins, J. (2019). RoB 2: A revised tool for assessing risk of bias in randomised trials. BMJ, 366(4898), 1–8. 10.1136/bmj.l4898 31462531

[cl21272-bib-0046] Sweet, E. , Nandi, A. , Adam, E. , & McDade, T. (2013). The high price of debt: household financial debt and its impact on mental and physical health. Social Science & Medicine, 91, 94–100. 23849243 10.1016/j.socscimed.2013.05.009 PMC3718010

[cl21272-bib-0047] Szubert, Z. , & Sobala, W. (2005). Current determinants of early retirement among blue collar workers in Poland. International Journal of Occupational Medicine and Environmental Health, 18(2), 177–184. 16201209

[cl21272-bib-0048] Sánchez‐Meca, J. , Marin‐Martinez, F. , & Chacon‐Moscoso, S. (2003). Effect‐size indices for dichotomized outcomes in meta‐analysis. Psychological Methods, 8(4), 448–467. 14664682 10.1037/1082-989X.8.4.448

[cl21272-bib-0049] Tanner‐Smith, E. E. , & Tipton, E. (2014). Robust variance estimation with dependent effect sizes: Practical considerations including a software tutorial in stata and SPSS. Research Synthesis Methods, 5, 13–30. 26054023 10.1002/jrsm.1091

[cl21272-bib-0050] Tipton, E. (2015). Small sample adjustments for robust variance estimation with meta‐regression. Psychological Methods, 20(3), 375–393. 24773356 10.1037/met0000011

[cl21272-bib-0051] Van De Voorde, K. , Paauwe, J. , & Van, V. M. (2012). Employee well‐being and the HRM‐organizational performance relationship: A review of quantitative studies. International Journal of Management Reviews, 14(4), 391–407.

[cl21272-bib-0052] WHO . (2000). Women's mental health: An evidence based review . WHO Report, pp. 1–121.

