# Supplementary material for: PROTOCOL: Performance pay and employee health: A systematic review
Source: Campbell Syst Rev. 2022 Aug 9;18(3):e1272. doi: 10.1002/cl2.1272 (PMC9363902; doi:10.1002/cl2.1272)
Supplement: Supplementary file 1 — Supporting information. [file CL2-18-e1272-s001.docx]

# Appendices

## 1 First and second level screening

First level screening is on the basis of titles and abstracts. Second level is on the basis of full text

Reference id. No. :

Reviewers initials:

Source:

Year of publication:

Country/countries of origin:

Author(s):

The study will be excluded if one or more of the answers to question 1-3 are ‘No’. If the answers to question 1 to 3 are ‘Yes’ or ‘Uncertain’, then the full text of the study will be retrieved for second level eligibility. All unanswered questions need to be posed again on the basis of the full text. If not enough information is available, or if the study is unclear, the author of the study will be contacted if possible.

**Screening questions:**

1. Does the study focus on performance payment?

Yes - include

No – if no then stop here and exclude

Uncertain - include

Question 1 guidance:

The intervention in this review is performance payment in contrast to payment per hour. Performance payment includes piece rates, bonuses, commissions, stock options and tips. The level of remuneration could be both at individual level (single worker) and group level (e.g. teams, plants or firms).

2. Does the study include an assessment of the impact of performance pay on the health of workers.

Yes - include

Uncertain - include

Question 2 guidance:

The primary outcomes in the study are measures related to the health of workers. These outcomes include:

- Accidents and injuries
- Physical and mental health outcomes (e.g. musculoskeletal problems, premature aging, stress, anxiety, depression etc.)
- Medicine use
- Use of health care resources (e.g., doctors visits, emergency department use),
- Sickness absence (or absenteeism) from the work-place,
- Early retirement
- Physiological effects (e.g. levels of measured adrenaline)

3. Is the report/article a quantitative evaluation study with a comparison group?

Yes - include

No – if no then stop here and exclude

Uncertain - include

Question 3 guidance:

We are only interested in quantitative studies with a comparison group, where the authors have analysed the data. We are not interested in theoretical papers on the topic or surveys/reviews of studies of the topic. (This question may be difficult to answer on the base of titles and abstracts alone.)

## 2 Data extraction

| Authors |
| --- |
| Title |
| Language |
| Journal |
| Year |
| Country |
| Industry |
| Type of performance pay (piece rates, commissions, bonuses, profit sharing etc.) |
| Unit of performance pay |
| Share of pay made up by performance pay |
| Participant characteristic (age, gender, occupation) |
| Length of exposure to performance pay |
| Time of measurement after exposure to performance pay |
| Type of data used in study (administrative, questionnaire, other (specify)) |
| Sample size (Treatment and control) |
| Research design (panel / cross-section data) |

**Outcome measures**

Instructions: Please enter outcome measures in the order in which they are described in the report. Note that a single outcome measure can be completed by multiple sources and at multiple points in time (data from specific sources and time-points will be entered later).

| # | Outcome  & measure | Reliability & Validity | Format | Direction | Pg# & notes |
| --- | --- | --- | --- | --- | --- |
| 1 |  | Info from:  Other samples  This sample  Unclear  Info provided: | Dichotomy  Continuous | High score or event is  Positive  Negative  Can’t tell |  |

* Repeat as needed

**OUT COME DATA**

**DICHOTOMOUS OUTCOME DATA**

| OUTCOME | TIME POINT (s) (record exact time from participation, there may be more than one, record them all) | SOURCE | VALID Ns | CASES | NON-CASES | STATISTICS | Pg. # & NOTES |
| --- | --- | --- | --- | --- | --- | --- | --- |
|  |  | Questionnaire  Admin data  Other (specify)  Unclear | Participation | Participation | Participation | RR (risk ratio)  OR (odds ratio)  SE (standard error)  95% CI  DF  P- value (enter exact p value if available)  Chi2  Other |  |
|  |  |  |  |  |  |  |  |
|  |  |  | Comparison | Comparison | Comparison |  |  |
|  |  |  |  |  |  |  |  |

Repeat as needed

**CONTINUOUS OUTCOME DATA**

| OUTCOME | TIME POINT (s) (record exact time from participation, there may be more than one, record them all) | SOURCE  (specify) | VALID Ns | Means | SDs | STATISTICS | Pg. # & NOTES |
| --- | --- | --- | --- | --- | --- | --- | --- |
|  |  | Questionnaire  Admin data  Other (specify)  Unclear | Participation | Participation | Participation | P  t  F  Df  ES  Other |  |
|  |  |  |  |  |  |  |  |
|  |  |  | Comparison | Comparison | Comparison |  |  |
|  |  |  |  |  |  |  |  |

*Repeat as need

## 3 Author Declaration

#### Authors’ responsibilities

By completing this form, you accept responsibility for preparing, maintaining and updating the review in accordance with Campbell Collaboration policy. Campbell will provide as much support as possible to assist with the preparation of the review.

A draft review must be submitted to the relevant Coordinating Group within two years of protocol publication. If drafts are not submitted before the agreed deadlines, or if we are unable to contact you for an extended period, the relevant Coordinating Group has the right to de-register the title or transfer the title to alternative authors. The Coordinating Group also has the right to de-register or transfer the title if it does not meet the standards of the Coordinating Group and/or Campbell.

You accept responsibility for maintaining the review in light of new evidence, comments and criticisms, and other developments, and updating the review at least once every five years, or, if requested, transferring responsibility for maintaining the review to others as agreed with the Coordinating Group.

#### Publication in the Campbell Library

The support of the Coordinating Group in preparing your review is conditional upon your agreement to publish the protocol, finished review, and subsequent updates in the Campbell Library. Campbell places no restrictions on publication of the findings of a Campbell systematic review in a more abbreviated form as a journal article either before or after the publication of the monograph version in Campbell Systematic Reviews. Some journals, however, have restrictions that preclude publication of findings that have been, or will be, reported elsewhere and authors considering publication in such a journal should be aware of possible conflict with publication of the monograph version in Campbell Systematic Reviews. Publication in a journal after publication or in press status in Campbell Systematic Reviews should acknowledge the Campbell version and include a citation to it. Note that systematic reviews published in Campbell Systematic Reviews and co-registered with Cochrane may have additional requirements or restrictions for co-publication. Review authors accept responsibility for meeting any co-publication requirements.

**I understand the commitment required to undertake a Campbell review, and agree to publish in the Campbell Library. Signed on behalf of the authors**:

| **Form completed by:** Karsten Albæk | **Date:** October 15, 2020 |
| --- | --- |
